# A Novel Hypersonic Target Trajectory Estimation Method Based on Long Short-Term Memory and a Multi-Head Attention Mechanism

**DOI:** 10.3390/e26100823

**Published:** 2024-09-26

**Authors:** Yue Xu, Quan Pan, Zengfu Wang, Baoquan Hu

**Affiliations:** 1 School of Automation, Northwestern Polytechnical University, Xi’an 710129, China; quanpan@nwpu.edu.cn (Q.P.); wangzengfu@nwpu.edu.cn (Z.W.); 2 School of Mechanical and Electrical Engineering, Lanzhou University of Technology, Lanzhou 730050, China; hubaoquan@lut.edu.cn; 3 School of Engineering, Xi’an International University, Xi’an 710077, China

**Keywords:** near space, hypersonic speed, target tracking, information entropy, information optimization

## Abstract

To address the complex maneuvering characteristics of hypersonic targets in adjacent space, this paper proposes an LSTM trajectory estimation method combined with the attention mechanism and optimizes the model from the information-theoretic perspective. The method captures the target dynamics by using the temporal processing capability of LSTM, and at the same time improves the efficiency of information utilization through the attention mechanism to achieve accurate prediction. First, a target dynamics model is constructed to clarify the motion behavior parameters. Subsequently, an LSTM model incorporating the attention mechanism is designed, which enables the model to automatically focus on key information fragments in the historical trajectory. In model training, information redundancy is reduced, and information validity is improved through feature selection and data preprocessing. Eventually, the model achieves accurate prediction of hypersonic target trajectories with limited computational resources. The experimental results show that the method performs well in complex dynamic environments with improved prediction accuracy and robustness, reflecting the potential of information theory principles in optimizing the trajectory prediction model.

## 1. Introduction

With the rapid development of aviation technology, hypersonic aircraft have shown enormous potential for applications in military reconnaissance, rapid strikes, and civilian transportation due to their unique speed and maneuverability [1,2]. However, the high speed and maneuverability of hypersonic aircraft also pose significant challenges in trajectory estimation. Traditional trajectory estimation methods, such as dynamic and kinematic models, often require a large amount of prior information and find it difficult to accurately capture the complex dynamic characteristics of hypersonic aircraft during flight [3]. Therefore, developing a new method that can accurately estimate the trajectory of hypersonic targets is of great significance for improving the efficiency of tasks such as target tracking, early warning, and interception [4].

At present, research on trajectory estimation of complex maneuvering targets mainly focuses on multi-model frameworks. To meet various estimation needs, researchers have continuously improved and optimized the Interactive Multiple Model (IMM) method from various aspects such as the selection of maneuvering models, the design of filtering algorithms, and the determination of weighting strategies between models. For example, He et al. [5] combined virtual maneuvering noise with first-order Markov process models and proposed a hypersonic target tracking method with adaptive corrected unbiased minimum variance estimation. Wu et al. [6] proposed a target tracking method based on multi-hypothesis fuzzy matching to address the issue of distance ambiguity in detecting weak targets in high pulse repetition rate radar. Li et al. [7] proposed a new target-tracking mode to address the difficulty of ground radar in effectively defending hypersonic aircraft. By dividing the tracking task into several tracking intervals with the same duration, and then dispatching multiple satellites for tracking within each interval, high tracking accuracy has been achieved. Huang et al. [8] achieved adaptive state estimation through unscented Kalman filtering, achieving stronger robustness and higher estimation accuracy. Li et al. [9] proposed a target tracking method based on adaptive kernel learning Kalman filtering. This method introduces the maximum entropy criterion and can update the state transfer function in real-time, achieving high tracking accuracy and convergence speed. Huang et al. [10] proposed a tracking method for hypersonic aircraft based on UKF filters, which solves the prediction error problem caused by aircraft tilt reversal maneuvers. Tang et al. [11] proposed a composite control method based on adaptive dynamic programming for contour tracking of hypersonic aircraft under multiple constraints. Liu et al. [12] conducted a detailed numerical simulation study on different maneuvering modes of hypersonic targets, and based on the simulation results, innovatively designed a finite time disturbance observer. This observer can accurately predict the acceleration information of hypersonic targets during flight, providing strong support for subsequent trajectory estimation and target tracking. Yang et al. [13] proposed a Multi-Granularity Scene Understanding framework (MGSU) integrating the Transformer structure, which significantly improves the accuracy of future trajectory prediction in complex scenarios through multi-granularity fusion and inverse reinforcement learning path generation. Although Transformer performs well in handling sequential data, LSTM may be a more appropriate choice when the amount of data is small, or the computational resources are limited. Due to LSTM’s lower model complexity and number of parameters, it is easier to train on small datasets and does not require as much computational resources as a Transformer. In addition, LSTM may be preferred in application scenarios such as hypersonic trajectory prediction where real-time requirements are very high. Because the inference process of LSTM is relatively simple and predictable, it is easier to meet real-time requirements.

However, despite significant progress in the field of trajectory estimation using multi-model frameworks, there are still many challenges when dealing with complex maneuvering targets, especially those with extremely high speeds and complex maneuverability such as hypersonic aircraft [14]. This requires trajectory estimation methods to have higher adaptability and robustness to cope with the complex behavioral characteristics exhibited by targets in dynamic environments [15].

Recently, the rapid development of deep learning [16] technology has provided new ideas for trajectory estimation. Especially the Long Short-Term Memory (LSTM) network [17], as a special type of Recurrent Neural Network (RNN) [18], has shown excellent performance in processing time series data due to its unique memory mechanism and gating unit [19]. LSTM can effectively capture long-term dependencies in time series data and exhibits good generalization ability when dealing with high-dimensional and nonlinear data [20]. Therefore, trajectory estimation methods based on LSTM have gradually become a research hotspot [21,22,23].

At present, some scholars have applied LSTM to the field of trajectory estimation and have achieved certain results. For example, Bartusiak et al. [24] proposed a machine-learning method for predicting the trajectory of maneuvering targets. Specifically, this method predicts the transition mode of the trajectory through random syntax, thereby predicting the kinematic characteristics of the aircraft. The proposed method was validated on two datasets, and the results showed that it can accurately predict the flight trajectory of the aircraft even in noisy environments. Xu et al. [25] proposed a multi-agent trajectory collaborative prediction model based on a Social Long Short-Term Memory (S-LSTM) network, which captures the interactions between aircraft by establishing an LSTM network for each plane and integrating the hidden states of associated aircraft, thereby improving the accuracy of trajectory predictions and the efficiency of airspace management. Pang et al. [26] introduced a weather-related trajectory prediction model that combines RNNs with convolutional layers, significantly reducing flight prediction bias and variance while enhancing management efficiency and safety during convective weather. Schimpf et al. [27] explored methods for 4D flight trajectory prediction using deep learning techniques, including LSTM, GRU, and RNN, in conjunction with various data sources. By incorporating attention mechanisms, they improved prediction accuracy. Song et al. [28] proposed a dynamic tracking algorithm based on LSTM to solve the problem of real-time tracking of maneuvering targets in complex electromagnetic environments. Firstly, LSTM is used to learn the motion characteristics of the target from the input noise data, and then the learned features are integrated into the proposed tracking algorithm, effectively capturing the motion trajectory of small and weak maneuvering targets. Li et al. [29] proposed a multi-target tracking method based on a bidirectional LSTM network. Firstly, the network is trained using offline multi-target data, and then the trained LSTM network is used for multi-target matching. Dai et al. [30] proposed a target trajectory tracking method based on an LSTM network, which learns and extracts the target’s motion pattern or model from the historical trajectory data of the target through the LSTM network. Subsequently, to further improve the accuracy and dynamic response ability of state estimation, the Kalman filtering algorithm was used to dynamically adjust and optimize the trajectory state predicted by the LSTM network in real-time. This combination enables the trajectory tracking system to learn the complex motion laws of the target while maintaining robustness in the face of uncertainty or noise. Yang et al. [31] proposed an innovative pedestrian trajectory prediction method called GTPPO, which integrates LSTM-encoded temporal attention movement patterns, graph attention-based social interaction capture, and uncertainty handling for multi-modal outputs. GTPPO demonstrated outstanding performance across multiple datasets, particularly excelling in handling sudden changes in movement, showcasing state-of-the-art prediction capabilities. However, these studies mainly focus on the trajectory estimation of low-speed or medium-speed targets, and there is relatively little research on the trajectory estimation of hypersonic targets. Hypersonic aircraft have extremely high speeds and complex maneuverability during flight, and their trajectory data often exhibits high nonlinearity and uncertainty, which poses greater challenges to LSTM-based trajectory estimation methods.

To solve the above problems, this paper proposes a novel hypersonic target trajectory estimation method based on LSTM and multi-head attention mechanism. It aims to accurately capture the dynamic characteristics of hypersonic vehicles during flight and realize the accurate prediction of target trajectories. The main contributions of this paper are as follows:

(1) In trajectory estimation, the motion trajectories of targets exhibit significant long-term dependencies, and these dependencies contain rich information. The LSTM network proposed in this paper can effectively capture and model this long-term dependency information through its unique structural advantages, to accurately predict the target’s trajectory.
(2) Traditional LSTM models usually assume that all input information is of equal importance, i.e., given the same weight, when dealing with time series data. However, in the complex scenario of hypersonic target trajectory estimation, data at different time points carry different amounts of information, which naturally have different impacts on the final estimation results. The LSTM model incorporating the attention mechanism proposed in this paper can dynamically assess the importance of the data based on its information entropy and automatically assign weights accordingly.
(3) Aiming at the real-time demand of hypersonic target trajectory estimation, this paper carries out an in-depth study on model optimization. By introducing a regularization term based on information gain, we reduce the model’s dependence on redundant information and improve the efficiency of information utilization. Meanwhile, a dynamic learning rate adjustment mechanism is designed, which can intelligently adjust the learning rate according to the change of information entropy during the training process and accelerate the convergence process of the model.

The remaining chapters of this paper are organized as follows: Section 2 briefly introduces the basic theory of LSTM and hypersonic vehicles; Section 3 describes the proposed trajectory estimation method; Section 4 validates the effectiveness of the proposed method; and, finally, the conclusion is given in Section 5.

## 2. Theoretical Foundations

### 2.1. Long Short-Term Memory (LSTM) Network

**Figure 1:**
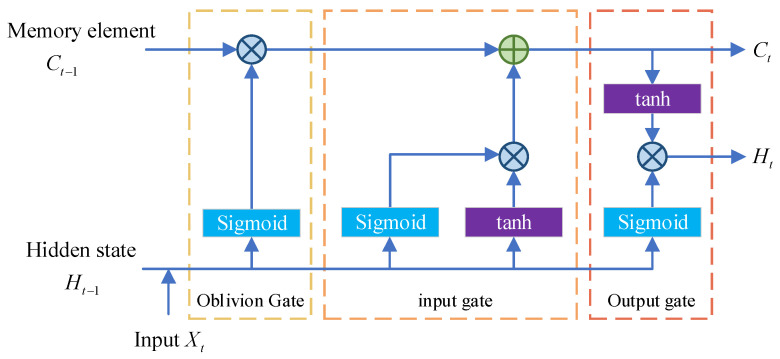
Basic structure of LSTM.

LSTM mainly consists of an oblivion gate, input gate, and output gate as shown in Figure 1. Its calculation formula is as follows:

(1) $$\begin{array}{l} I_{t}=\mathrm{sigmoid}\left(X_{t}W_{xi}+H_{t-1}W_{hi}+b_{i}\right)\\ F_{t}=\mathrm{sigmoid}\left(X_{t}W_{xf}+H_{t-1}W_{hf}+b_{f}\right)\\ O_{t}=\mathrm{sigmoid}\left(X_{t}W_{xo}+H_{t-1}W_{ho}+b_{o}\right) \end{array}$$

where $I_{t}\in {\mathbb{R}}^{n\times h}$ is the input gate, $F_{t}\in {\mathbb{R}}^{n\times h}$ is the forgetting gate, $O_{t}\in {\mathbb{R}}^{n\times h}$ is the output gate, $W_{xi},W_{xf},W_{xo}\in {\mathbb{R}}^{d\times h}$ and $W_{hi},W_{hf},W_{ho}\in {\mathbb{R}}^{h\times h}$ are the weighting coefficients, $b_{i},b_{f},b_{o}\in {\mathbb{R}}^{1\times h}$ is the bias parameter, and sigmoid is the activation function, which is calculated as follows:

(2) $$\mathrm{sigmoid}(x)=\frac{1}{1+e^{-x}}$$

The formula for candidate memory cell ${\tilde{C}}_{t}\in {\mathbb{R}}^{n\times h}$ at time step t is as follows:

(3) $${\tilde{C}}_{t}=\tanh\left(X_{t}W_{xc}+H_{t-1}W_{hc}+b_{c}\right)$$

where $W_{xc}\in {\mathbb{R}}^{d\times h}$ and $W_{hc}\in {\mathbb{R}}^{h\times h}$ are the weighting coefficients and $b_{c}\in {\mathbb{R}}^{1\times h}$ is the bias parameter.

In LSTM, the input gate $I_{t}$ controls how much new data from ${\tilde{C}}_{t}$ is employed, while the forget gate $F_{t}$ controls how much of the content of the past memory element $C_{t-1}\in {\mathbb{R}}^{n\times h}$ is retained, as obtained in the following:

(4) $$C_{t}=F_{t}*C_{t-1}+I_{t}*{\tilde{C}}_{t}$$

The hidden state $H_{t}\in {\mathbb{R}}^{n\times h}$ is a gated version of the tanh of the memory element, ensuring that the value of $H_{t}$ is in the interval (−1,1):

(5) $$H_{t}=O_{t}*\tanh\left(C_{t}\right)$$

where tanh is the hyperbolic tangent function, calculated as follows:

(6) $$\tanh\left(x\right)=\frac{e^{x}-e^{-x}}{e^{x}+e^{-x}}$$

### 2.2. Dynamic Model of Hypersonic Aircraft

The motion equation of a conventional hypersonic aircraft in a ballistic coordinate system [32] is:

(7) $$\left\{\begin{array}{l} \dot{x}=v\cos\gamma \sin\psi \\ \dot{y}=v\cos\gamma \cos\psi \\ \dot{z}=v\sin\gamma \\ \dot{v}=-\frac{D}{m}-g\sin\gamma \\ \dot{\gamma }=\frac{L\cos\sigma }{mv}-\frac{g}{v}\cos\gamma \\ \dot{\psi }=\frac{L\sin\sigma }{mv\cos\gamma } \end{array}\right.$$

where v and m are the flight speed and mass of the hypersonic target, respectively; γ is the ballistic inclination (the angle between the velocity vector and the horizontal plane); ψ is the ballistic inclination (the angle between the projection of the velocity vector in the horizontal plane and the y-axis); g is the acceleration of gravity, taken as 9.81 $m/s^{2}$; σ is the tilt angle; L is the aerodynamic lift; and D is the aerodynamic drag. The expressions for *L* and *D* are as follows:

(8) $$\begin{array}{l} L=\frac{1}{2}\rho \left(z\right)v^{2}C_{L}\left(\alpha ,Ma\right)S\\ D=\frac{1}{2}\rho \left(z\right)v^{2}C_{D}\left(\alpha ,Ma\right)S \end{array}$$

where $\rho \left(z\right)$ is the air density versus altitude curve; Ma is the flight Mach number; α is the angle of attack; and S is the vehicle characteristic area.

## 3. Proposed Methods

Traditional methods for predicting the trajectories of hypersonic targets often struggle to accurately capture dynamic changes in the target and overlook important feature information, leading to insufficient prediction accuracy and poor robustness. To overcome these challenges, we propose an LSTM trajectory estimation method integrated with a multi-head attention mechanism. This approach leverages the strengths of LSTM in sequence modeling and combines it with a multi-head attention mechanism, allowing for dynamic adjustment of attention to different historical trajectory information, effectively extracting key features for precise recognition and prediction of target motion behavior. This design not only improves prediction accuracy but also enhances the model’s adaptability to complex dynamic characteristics, providing more reliable technical support for monitoring and tracking hypersonic targets in near space. In this section, we will detail the proposed method, including the LSTM network integrated with the attention mechanism, the multi-head attention mechanism, model parameters, and the network training process.

### 3.1. LSTM Network Incorporating Attention Mechanism

For the overall structural design of the network, we integrate the algorithmic essence of the multi-head attention mechanism and LSTM, and this design aims to achieve a dual objective: firstly, to enhance the recognition accuracy by enhancing the model’s ability to capture the intrinsic relevance and complexity of the data; and secondly, to take advantage of the complementary nature of the two mechanisms to optimize the learning process, thus accelerating the network’s convergence speed.

**Figure 2:**
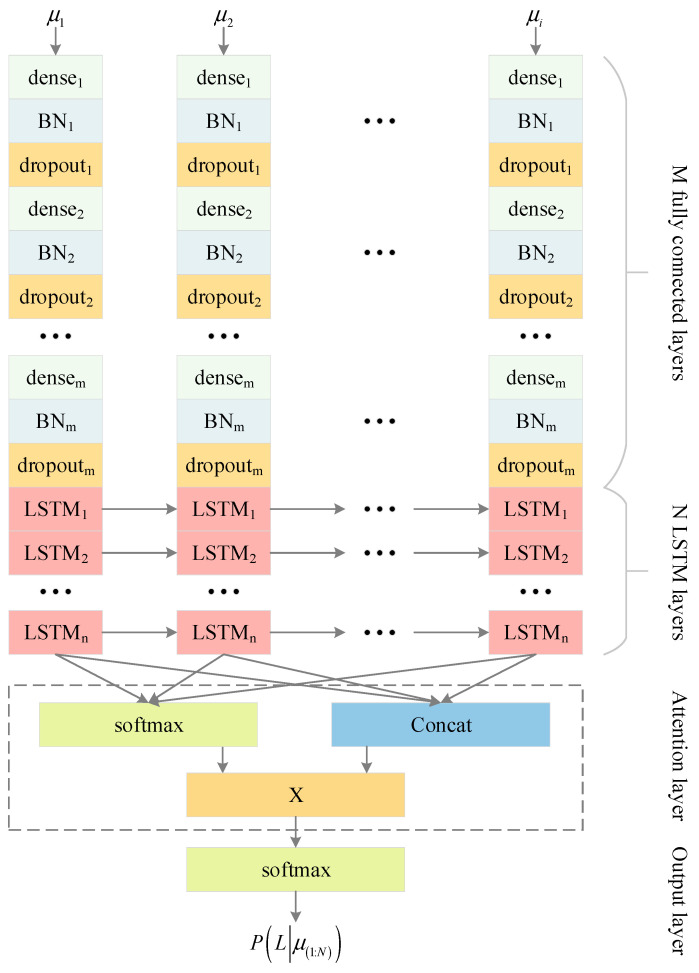
LSTM network incorporating attention mechanism.

Figure 2 shows a schematic diagram of the network structure. Considering the relatively low dimensionality of the input data, we first use M fully connected layers (Dense layers) to perform dimensionality enhancement operations, thereby enriching the feature representation of the input data. Through this dimensionality enhancement step, we can ensure that subsequent network layers can more effectively process and parse these enhanced features. The fully connected layer is represented as:

(9) $$\delta =\mathrm{Dense}\left(\mu \right)=\tanh\left(wx+b\right)$$

When constructing the network structure, we particularly focused on the parameter configuration and regularization methods of the fully connected layer (Dense layer) to optimize the performance of the network. The weights w and biases b of the fully connected layer are key parameters for model learning, which are updated during the training process to minimize prediction errors.

To prevent overfitting and improve the generalization ability of the network, we have introduced two effective regularization techniques: batch normalization (BN) and dropout.

The BN layer is represented as:

(10) $$\begin{array}{l} {\hat{x}}_{i}=\frac{x_{i}-\bar{\mu_{B}}}{\sqrt{{\hat{\sigma }}_{B}^{2}+\varepsilon }}\\ \bar{\mu_{B}}=\frac{1}{\left|B\right|}\sum_{x\in B}x\\ {\hat{\sigma }}_{B}^{2}=\frac{1}{\left|B\right|}{\sum_{x\in B}\left(x-\bar{\mu_{B}}\right)}^{2}+\varepsilon \end{array}$$

where x is the input data; $\bar{\mu_{B}}$ and ${\hat{\sigma }}_{B}^{2}$ are the mean and variance of the input data, respectively; ε>0 is a constant; and ${\hat{x}}_{i}$ is the data obeying the standard normal distribution after transformation.

Through Formula (10), the input data x, which was originally randomly distributed, can be transformed into data ${\hat{x}}_{i}$ that satisfies a normal distribution, thereby making the distribution of data in the input network closer, which is conducive to the iterative updating of the network.

The LSTM network structure with BN layer and attention mechanism is shown in Figure 2, and its mathematical expression is as follows:

(11) $$\left\{\begin{array}{l} \delta_{k,1}=Dense_{1}\left(\mu_{k}\right)\\ {\bar{\delta }}_{k,1}=BN_{1}\left(\delta_{k,1}\right)\\ {\overset{⏜}{\delta }}_{k,1}=Dropout_{1}\left({\bar{\delta }}_{k,1}\right)\\ \delta_{k,j}=Dense_{j}\left({\overset{⏜}{\delta }}_{k,j-1}\right)\\ {\bar{\delta }}_{k,j}=BN_{j}\left(\delta_{k,j}\right)\\ {\overset{⏜}{\delta }}_{k,j}=Dropout_{j}\left({\bar{\delta }}_{k,j}\right)\\ \left[\delta_{k,LSTM_{1},}C_{k,LSTM_{1}}\right]=LSTM_{1}\left(\delta_{K-1,LSTM_{1}},C_{k-1,LSTM_{1}},{\overset{⏜}{\delta }}_{k,d}\right)\\ \left[\delta_{k,LSTM_{i},}C_{k,LSTM_{i}}\right]=LSTM_{i}\left(\delta_{K-1,LSTM_{i}},C_{k-1,LSTM_{i}},\delta_{k,LSTM_{\left(i-1\right)}}\right)\\ \delta_{\left(1:N\right),LSTM_{l}}=\left[\delta_{1,LSTM_{l}},\delta_{2,LSTM_{l}},\ldots ,\delta_{N,LSTM_{l}}\right]\\ w_{att}=\mathrm{Softmax}\left(w_{att1}\delta_{\left(1:N\right),LSTM_{l}}+b_{att}\right)\\ \delta_{att}=w_{att}^{T}\left(\delta_{\left(1:N\right),LSTM_{l}}\right)\\ P\left(Labels\left|\mu \left(1:N\right)\right.\right)=\mathrm{Softmax}\left(\delta_{att}\right) \end{array}\right.$$

where i=1,2,…,n, j=2,3,…,m, k=1,2,…,N.

### 3.2. Multi-Attention Mechanisms

The self-attention mechanism is a sequence processing method that enhances the representation and performance of the model by generating query, key, and value vectors for each sequence element, calculating the relevance weights of each element with respect to the other elements in the sequence, and applying these weights to the corresponding value vectors in a weighted summation that dynamically focuses on the part of the sequence that is the most relevant to the task at hand.

**Figure 3:**
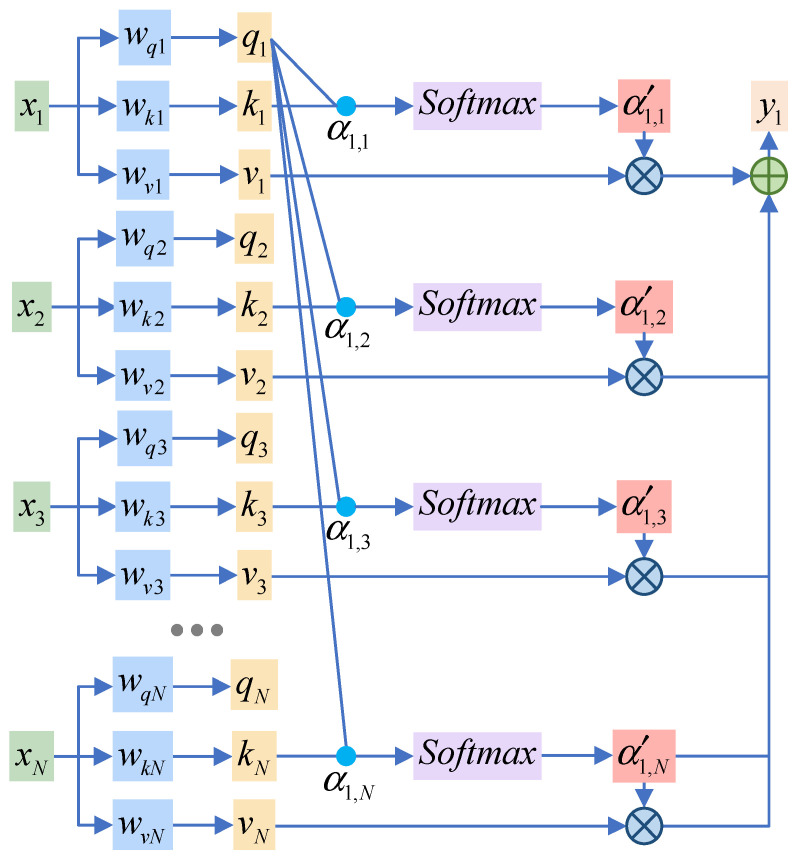
Self-attention module.

The self-attention mechanism is shown in Figure 3 and is calculated as follows:

(12) $$\left\{\begin{array}{c} q_{i}=w_{qi}*x_{i}\\ k_{i}=w_{ki}*x_{i}\\ v_{i}=w_{vi}*x_{i} \end{array}\right.$$

where $x_{i}$ is the input sequence data; $w_{q}$, $w_{k}$, and $w_{v}$ are the weight matrices; and q, k, and v are the obtained feature representations.

Then, by using the feature representation obtained in the previous step, the matrix of query, key, and value can be calculated.

(13) $$\left\{\begin{array}{c} Q=W_{q}*X\\ K=W_{k}*X\\ V=W_{v}*X \end{array}\right.$$

where Q, K and V denote the query, key, and value matrices, respectively.

Next, we calculate the attention score:

(14) $$\alpha_{i,j}^{\prime }=Softmax(q_{i}⊙k_{i})$$

where ⊙ denotes the dot product of q and k. The SoftMax function is used to calculate the normalized weight coefficients.

Finally, multiply the attention score by v to obtain the output sequence y:

(15) $$y_{j}=\sum_{i=1}^{N}\alpha_{j,i}^{\prime }*v_{i}$$

**Figure 4:**
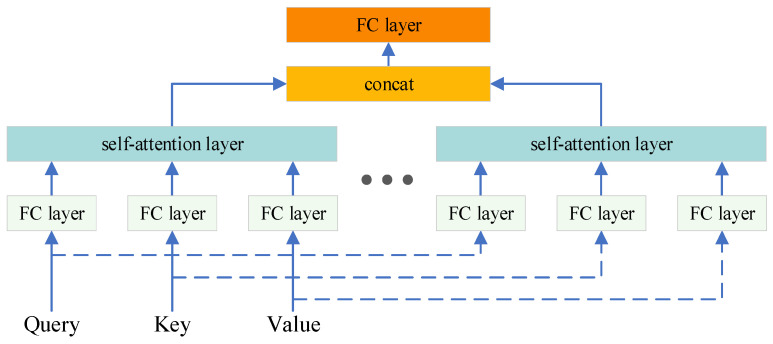
Multi-head attention module.

Multi-head attention, as shown in Figure 4, is an extended form of self-attention mechanism. The main advantage of multi-head attention is that it can process the input sequence with attention from different perspectives in parallel, thus improving the model’s ability to understand and capture complex dependencies.

### 3.3. Model Parameters

The structural parameters of each layer of the network are shown in Table 1, where the network parameters used are m=2 and n=2, which are two fully connected layers and two LSTM layers, with a neuron count of 256-128-128-256, respectively. To improve the performance of the model, the Dropout layer and BN layer were added to the fully connected layer. The Dropout layer randomly discards neurons, allowing the model to have different structures during each training session, thereby increasing the diversity of the model. The BN layer makes it easier for the network to learn the distribution of data and improve the model’s generalization ability through standardized operations.

The training parameters of the network are set as shown in Table 2. Specifically, the partition ratio between the training and testing sets is set to 8:2, where 80% of the input data is used as the training set and the remaining 20% is used as the testing set. The maximum number of iterations is 200. Choose a binary cross entropy loss function to calculate the model’s loss, select the Adam optimizer to update parameters in the network in real-time, and set the proportion of randomly discarded neurons in the Dropout layer to 0.2.

### 3.4. Network Training Process

**Figure 5:**
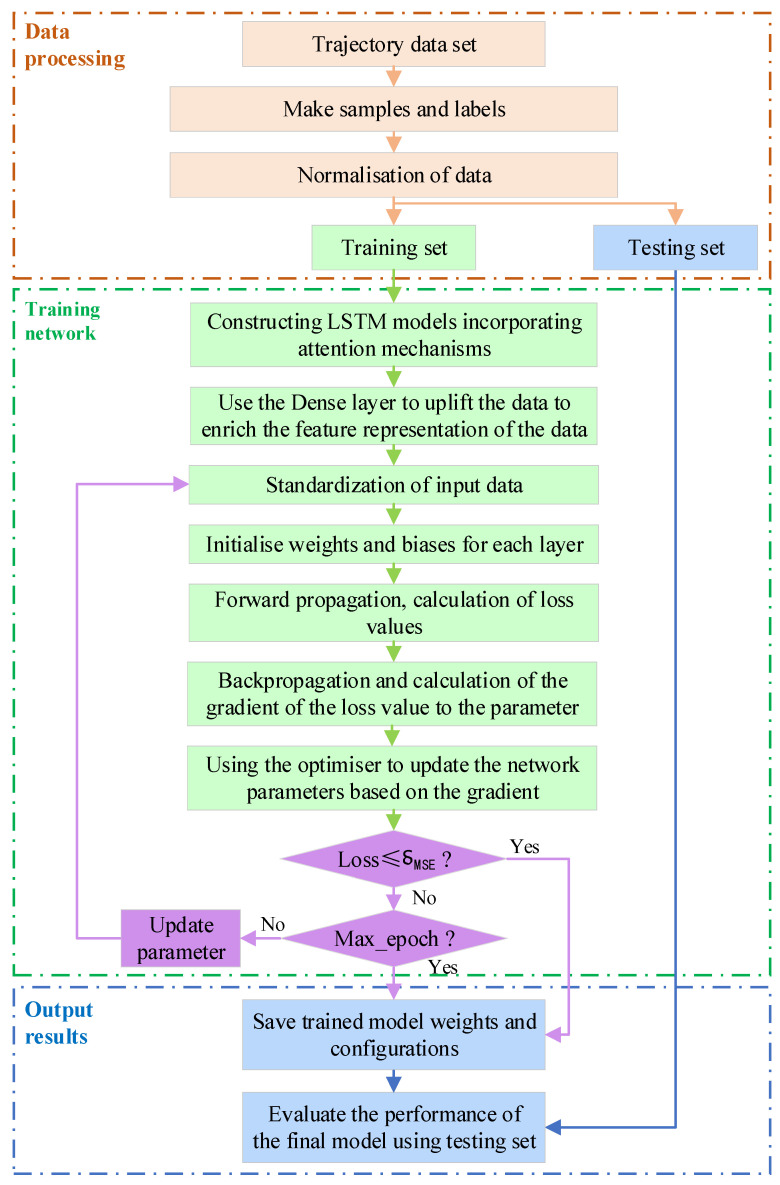
Network training flow.

Based on the LSTM model that integrates a multi-head attention mechanism, and in conjunction with the segmentation of trajectory data samples and the model testing phase, a complete trajectory prediction process has been designed, as shown in Figure 5. Below are more detailed descriptions of the main steps in this process:

(1) Perform preprocessing operations like standardization and normalization on the data to ensure it is on an appropriate scale for model training.
(2) Divide the preprocessed trajectory dataset into training and testing sets. The training set is used for model training, while the testing set is used to evaluate the model’s final performance.
(3) Use a sliding window technique to segment the trajectory data into multiple samples. Each sample contains trajectory data points over a period and their corresponding target variable (e.g., future position).
(4) Design and construct the model architecture, including multi-head attention layers to capture intrinsic relationships in the data and LSTM layers to capture dynamic features of the time series. Insert batch normalization (BN) and Dropout layers appropriately to enhance training efficiency and prevent overfitting. Set up the input and output layers to ensure the input data dimensions match the model and that the output layer can predict the target variable.
(5) Set initial weights and biases for each layer in the model, using either random initialization or pretrained weights.
(6) Input samples from the training set into the model for training, calculating the model’s output values. Compute the loss function (Mean Squared Error, MSE) based on the model’s output and true values. Use the Adam optimizer to compute the gradient of the loss function with respect to model parameters and update the parameters.
(7) When the model’s performance on the training set meets predefined stopping criteria (e.g., loss no longer significantly decreases, or a set number of iterations is reached), save the model’s weights and parameters.
(8) Evaluate the trained model using the testing set, calculating performance metrics on the test data.

## 4. Experimental Comparative Analysis

### 4.1. Description of the Data Set

The publicly released CAV-H model from Lockheed Martin [33] was used, which has a total mass of 907 kg and an aerodynamic reference area of 0.4839 m^2^. The aerodynamic coefficient wind tunnel test tables are shown in Table 3 and Table 4.

An equation fit to the aerodynamic data was used to replace the direct interpolation method for aerodynamic data with Mach numbers greater than 5 in Table 5 and Table 6.

$C_{L}$ is fitted to the angle of attack α and flight Mach number Ma as follows:

(16) $$\begin{array}{c} C_{L}\left(\alpha ,Ma\right)=-0.0561-0.00443Ma+0.05\alpha -0.00083Ma\cdot \alpha \\ \;\;\;\;\;\;\;\;\;\;\;\;+0.00032Ma^{2}+0.00037\alpha^{2} \end{array}$$

$C_{D}$ is fitted to the angle of attack α and flight Mach number Ma as follows:

(17) $$\begin{array}{c} C_{D}\left(\alpha ,Ma\right)=0.12721-0.01542Ma+0.00486\alpha -0.0003Ma\cdot \alpha \\ \;\;\;\;\;\;\;\;\;\;\;\;+0.00057Ma^{2}+0.00067\alpha^{2} \end{array}$$

By substituting the $C_{L}$ and $C_{D}$ obtained from Equations (16) and (17) into Formula (8) in Section 2.2, we can calculate the lift L and drag D. Then, substituting lift L and drag D into Formula (7) allows us to solve for various trajectory data. Using this method, we constructed a dataset containing 11,520 trajectory entries. This dataset not only includes basic flight patterns, such as quasi-equilibrium gliding and hopping gliding, but also incorporates complex target maneuver types, such as sharp turns, agile evasive actions, and periodic penetration strategies.

To verify and evaluate the performance of the machine learning models trained on these trajectory data, we randomly split the dataset into a training set and a testing set. Specifically, 80% of the trajectories were selected as the training set for the model’s training and learning process, while the remaining 20% served as the testing set to assess the model’s generalization ability and accuracy on unseen data.

### 4.2. Experimental Results

In order to validate the effectiveness of the proposed method, four different network architectures were selected for comparative analysis: (1) Case 1: LSTM combining batch normalization and a multi-head attention mechanism (Att-LSTM+BN); (2) Case 2: LSTM enhanced by a multi-head attention mechanism (Att-LSTM); (3) Case 3: LSTM combining batch normalization (LSTM+BN); and (4) Case 4: LSTM.

The network was trained using a computer with an AMD Ryzen 7 4800H CPU, Nvidia GeForce RTX 2060 GPU, and 32 GB of RAM, based on the Python 3.7 + TensorFlow 2.3 + Keras 2.7 platform.

**Figure 6:**
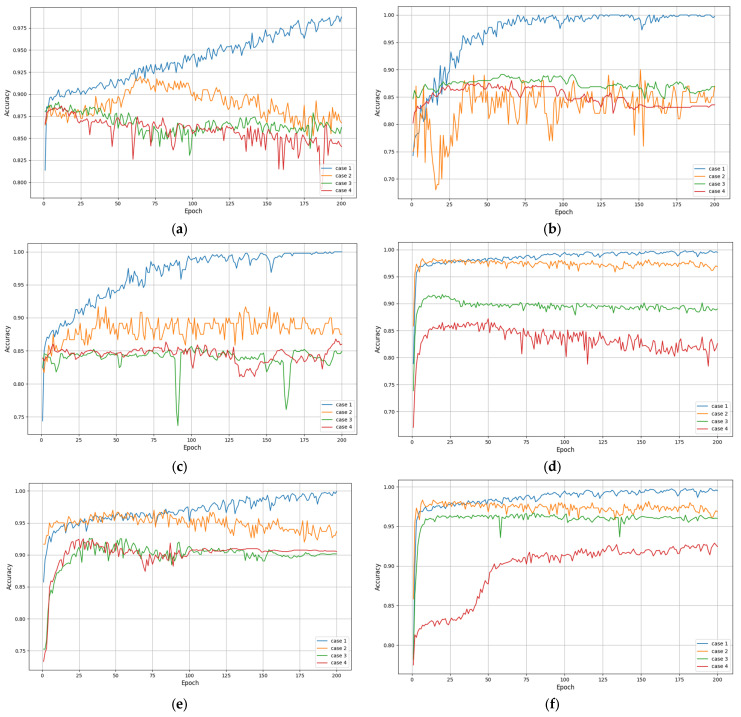
Recognition accuracy of various methods under different input sequences: (**a**) length = 50; (**b**) length = 100; (**c**) length = 150; (**d**) length = 200; (**e**) length = 250; (**f**) length = 300.

Since the input sequence length will have an impact on the recognition accuracy in the learning training of time series data. Therefore, we first analyze the effect of input sequence length on the recognition accuracy of the network. The network structure parameters used, m=2 and n=2, i.e., two fully connected layers and two LSTM layers, and the neurons number 256-128-128-256. The lengths of the input sequences are 50, 100, 150, 200, 250, and 300, respectively, and the experimental results are shown in Figure 5 and Figure 6. Accuracy and MSE were used as the evaluation metrics of the model with the following expressions:

(18) $$Accuracy=\frac{TP+TN}{TP+TN+FP+FN}$$

In the formula, TP (True Positive) represents the number of positive samples correctly classified as positive, TN (True Negative) indicates the number of negative samples correctly classified as negative, FP (False Positive) refers to the number of negative samples incorrectly classified as positive, and FN (False Negative) denotes the number of positive samples incorrectly classified as negative.

(19) $$MSE=\frac{1}{n}\sum_{i=1}^{n}{\left(y_{i}-{\hat{y}}_{i}\right)}^{2}$$

where n is the total number of samples, $y_{i}$ is the true value of the ith sample, and ${\hat{y}}_{i}$ is the predicted value of the ith sample.

Figure 6 and Table 5 show the recognition accuracy of four methods under six different input sequence lengths. As the input sequence length increases, the recognition accuracy of each method also increases. Moreover, when the length of the input sequence is greater than or equal to 200, the recognition accuracy of various methods becomes more stable. Furthermore, it can be observed that when the input sequence length is greater than or equal to 200, the two methods incorporating multi-head attention have higher and more stable recognition accuracy. Overall, the proposed method achieved the highest and most robust recognition accuracy under six different input sequences.

**Table 5:** Average recognition accuracy (%) of various methods with different input sequences.

Case	Length					
	50	100	150	200	250	300
Att-LSTM+BN	98.2	99.6	99.3	99.8	99.7	99.8
Att-LSTM	90.2	89.6	89.5	96.7	95.8	98.7
LSTM+BN	87.5	86.4	83.4	91.6	92.4	96.8
LSTM	85.3	83.6	82.4	86.4	91.6	93.9

**Figure 7:**
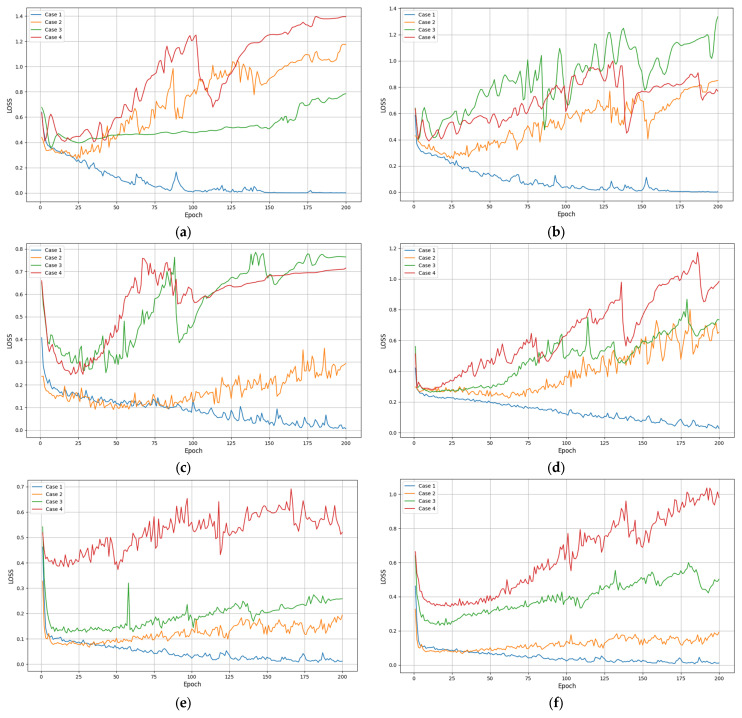
Recognition losses of various methods under different input sequences: (**a**) length = 50; (**b**) length = 100; (**c**) length = 150; (**d**) length = 200; (**e**) length = 250; (**f**) length = 300.

Figure 7 and Table 6 show the recognition loss of four methods under six input sequence lengths. The loss value is usually used to measure the prediction error of the model during the training process, while the accuracy directly reflects the classification accuracy of the model on the test set. Usually, the lower the loss value, the better the performance of the method. From Figure 7, the four methods exhibit lower loss values for all input sequence lengths. Relatively speaking, the loss value of our method significantly decreases with the increase in iteration times and remains relatively stable at different sequence lengths. This once again proves the effectiveness and robustness of the proposed method.

**Table 6:** Average loss of various methods with different input sequences.

Case	Length					
	50	100	150	200	250	300
Att-LSTM+BN	0.14	0.11	0.10	0.06	0.05	0.03
Att-LSTM	0.68	0.65	0.24	0.62	0.19	0.17
LSTM+BN	1.17	1.21	0.78	0.68	0.28	0.46
LSTM	1.39	0.97	0.76	1.17	0.67	0.78

When designing deep learning models based on LSTM, the number of LSTM layers and the configuration of fully connected layers have a crucial impact on network accuracy. The LSTM layer is responsible for capturing long-term dependencies in the input sequence, and different numbers of LSTM layers will affect the model’s ability to learn these dependencies.

Meanwhile, the role of fully connected layers in LSTM networks is to convert the output of LSTM layers into the final prediction results. The configuration of fully connected layers, including the number of layers and the number of neurons in each layer, also has a significant impact on the accuracy of the model. Too few fully connected layers or neurons may not fully utilize the features extracted by the LSTM layer, resulting in the model being unable to make accurate predictions. However, excessive fully connected layers or neurons may make the model too complex, increasing the risk of overfitting.

**Figure 8:**
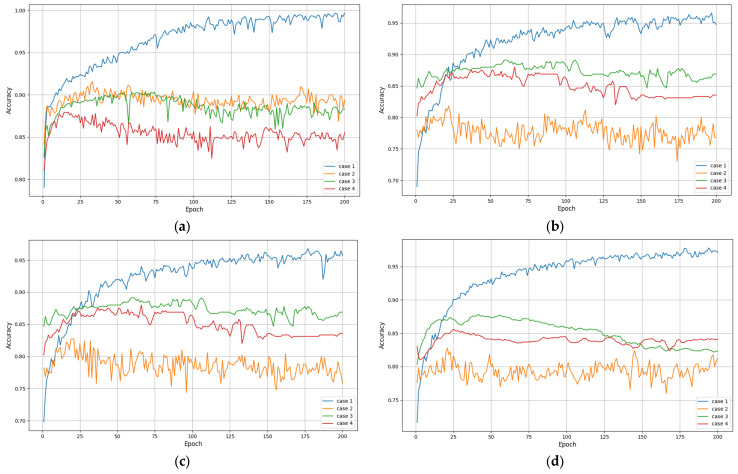

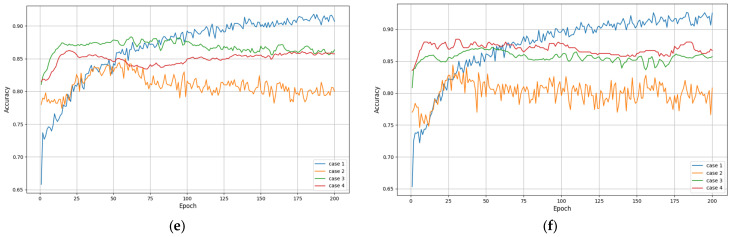
Recognition accuracy of various methods at different network layers: (**a**) m = 1, n = 1; (**b**) m = 1, n = 2; (**c**) m = 2, n = 1; (**d**) m = 2, n = 2; (**e**) m = 2, n = 3; (**f**) m = 3, n = 2.

**Figure 9:**
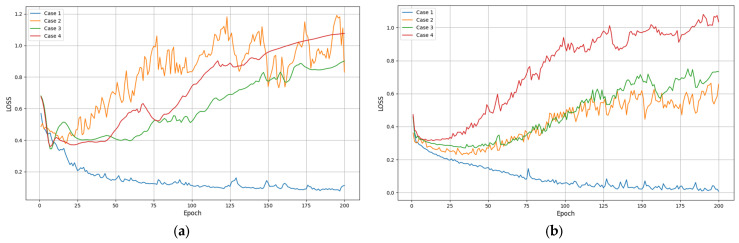

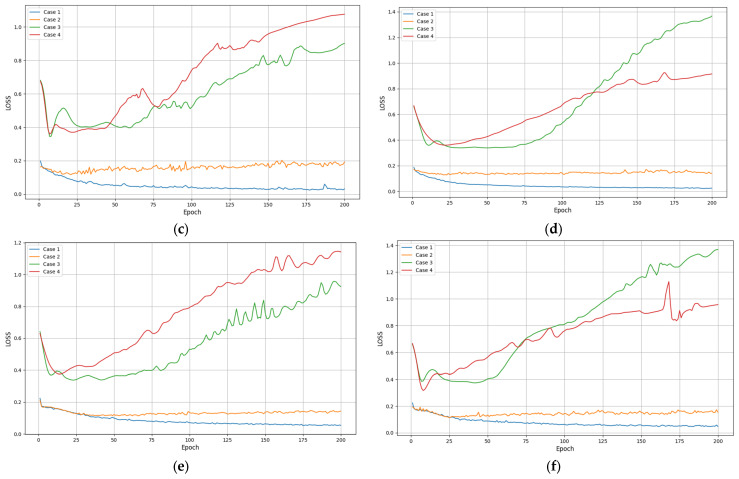
Recognition losses of various methods at different network layers: (**a**) m = 1, n = 1; (**b**) m = 1, n = 2; (**c**) m = 2, n = 1; (**d**) m = 2, n = 2; (**e**) m = 2, n = 3; (**f**) m = 3, n = 2.

In summary, to achieve optimal network accuracy, it is necessary to carefully consider the configuration of the LSTM layer and the fully connected layer. By exploring the combination of different layers and neuron numbers through experiments, combined with appropriate activation functions and regularization techniques, the most suitable model configuration for specific tasks and datasets can be found. Therefore, based on the network model designed in Figure 1, different sum values were used for experiments (i.e., using different fully connected layers and LSTM layers), and the experimental results are shown in Figure 8 and Figure 9, as well as Table 7 and Table 8.

From Figure 8 and Table 7, as the number of network layers increases, the recognition accuracy of various methods has increased. Relatively speaking, the proposed method has higher and more robust recognition accuracy. From Figure 9 and Table 8, the proposed method has lower and more robust losses, and when both the fully connected layer and LSTM reach more than two layers, the attention mechanism integrated into the model can demonstrate its effectiveness, which once again proves the effectiveness of the proposed method.

### 4.3. Diagnostic Performance Analysis under Noise Interference

To further validate the robustness and effectiveness of the proposed method in noisy environments, we introduced Gaussian white noise into the original dataset and tested it under different signal-to-noise ratio (SNR) [34] conditions. Specifically, we chose four representative scenarios, SNR = −6, −2, 2, and 6, to simulate a data environment from very noisy to relatively clean.

**Figure 10:**
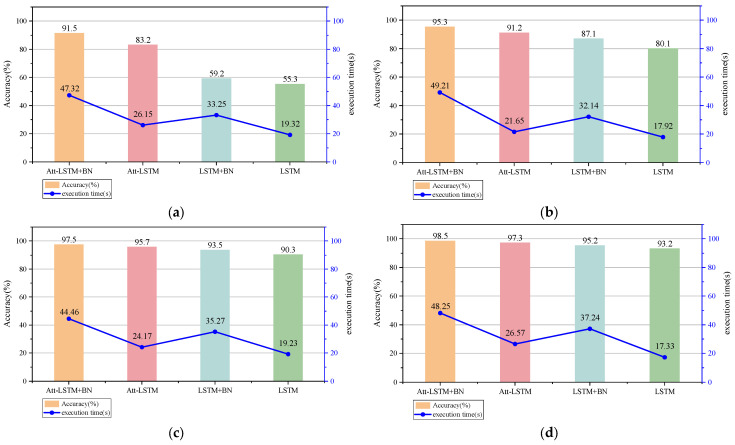
Performance of various methods in noisy environments: (**a**) SNR = −6; (**b**) SNR = −2; (**c**) SNR = 2; (**d**) SNR = 6.

The experimental results are shown in Figure 10, and the Att-LSTM+BN model exhibits the best performance in all four noise environments. This is mainly attributed to its combination of attention mechanism and batch normalization advantages, which enables the model to better focus on important information while effectively reducing internal covariate shifts, thereby improving stability and accuracy in noisy environments.

In contrast, although the Att-LSTM model also utilizes attention mechanisms to capture key information, its noise resistance is slightly insufficient without batch normalization. Similarly, although the LSTM+BN model improves its stability to some extent through batch normalization, it lacks guidance from attention mechanisms, resulting in limited performance in noisy environments. The standard LSTM model performs the worst in situations with high noise, mainly because it lacks attention mechanisms to focus on key information and batch normalization to reduce the impact of internal covariate shifts.

In summary, through experimental analysis under different SNR conditions, we have once again demonstrated the excellent accuracy of the Att-LSTM+BN model in processing noisy data, which further verifies the effectiveness and robustness of the model in complex noisy environments.

## 5. Conclusions

In this paper, we propose a novel trajectory estimation method that combines the temporal processing capability of LSTM networks with the multi-head information entropy attention mechanism, specifically for trajectory estimation of hypersonic targets. The fast maneuverability and high uncertainty of hypersonic targets in complex and changing flight environments make trajectory estimation a difficult task in aerospace.

By introducing the LSTM network, we successfully capture the temporal dependence and long-term memory information in the target trajectory, which provides the model with the ability to deeply understand the target’s movement patterns. The recurrent structure of LSTM allows the model to learn and retain historical information, which is crucial for predicting future trajectories.

However, relying on LSTM alone may not be enough to adequately cope with the complexity and uncertainty of hypersonic targets. Therefore, we further introduce a multi-head information entropy attention mechanism. This mechanism not only enhances the model’s attention to critical information, but also quantifies the information content and uncertainty of the data at different time points by calculating the information entropy. In this way, the model can intelligently assign weights and give higher attention to data with more information and lower uncertainty, thus improving the accuracy and robustness of trajectory estimation.

The experimental results show that the trajectory estimation method, which combines LSTM with the multi-head information entropy attention mechanism, exhibits significant advantages when dealing with hypersonic target trajectory data. Compared with the traditional method, this method shows a substantial improvement in both prediction accuracy and error rate. Especially when the target undergoes rapid maneuvers or is subject to external interference, the proposed method can still maintain stable performance, which fully proves its applicability and reliability in complex environments.

In addition, this method has good scalability and flexibility. By adjusting the network structure and parameter settings, we can further optimize the model performance to adapt to the needs of trajectory estimation in different scenarios. Meanwhile, information entropy, as an effective tool to quantify the importance of information, provides new ideas and methods for model optimization, which helps to promote the continuous progress of trajectory estimation technology.

## Figures and Tables

**Table 1:** Parameters of each layer in the network.

Layer	Number of Neurons	Output Shape	Param #
Dense_1	256	5 × 256	1280
BN	-	5 × 256	1024
Dropout	-	5 × 256	0
Dense_2	128	5 × 128	32,896
BN	-	5 × 128	512
Dropout	-	5 × 128	0
Multi Attention Module	-	5 × 128	0
LSTM_1	128	5 × 128	131,584
LSTM_2	256	5 × 256	394,240
Dense_3	1	5 × 1	257

**Table 2:** Setting of training parameters.

Parameters	Value
Training and testing set ratio	8:2
Maximum number of iterations	200
loss function	binary_crossentropy
optimizer	Adam
Dropping probability	0.2

**Table 3:** Lift coefficients ($C_{L}$).

α (°)	Ma 3.5	Ma 5	Ma 8	Ma 10	Ma 15	Ma 20	Ma 23
10	0.450	0.425	0.400	0.380	0.370	0.360	0.350
15	0.740	0.700	0.670	0.630	0.600	0.570	0.557
20	1.050	1.000	0.950	0.900	0.850	0.800	0.780

**Table 4:** Resistance coefficients ($C_{D}$).

α (°)	Ma 3.5	Ma 5	Ma 8	Ma 10	Ma 15	Ma 20	Ma 23
10	0.205	0.170	0.129	0.109	0.109	0.109	0.109
15	0.296	0.263	0.224	0.197	0.195	0.192	0.192
20	0.477	0.423	0.354	0.310	0.305	0.300	0.300

**Table 7:** Average recognition accuracy (%) of the various methods with different network layers.

Case	m = 1, n = 1	m = 1, n = 2	m = 2, n = 1	m = 2, n = 2	m = 2, n = 3	m = 3, n = 2
Att-LSTM+BN	97.9	96.8	95.8	96.7	93.6	97.8
Att-LSTM	89.6	79.6	80.6	80.6	82.6	85.2
LSTM+BN	86.8	86.3	88.7	86.4	87.9	86.7
LSTM	85.4	86.7	85.3	85.7	86.8	88.6

**Table 8:** Average loss of various methods with different network layers.

Case	m = 1, n = 1	m = 1, n = 2	m = 2, n = 1	m = 2, n = 2	m = 2, n = 3	m = 3, n = 2
Att-LSTM+BN	0.16	0.12	0.11	0.09	0.06	0.02
Att-LSTM	1.32	0.58	0.19	0.12	0.11	0.10
LSTM+BN	0.86	0.71	0.77	1.25	0.87	1.05
LSTM	0.93	1.08	0.99	0.89	1.26	0.98

## Data Availability

ADS-B datasets can be downloaded from https://flightadsb.variflight.com/track-data, accessed on 16 January 2024.
